# Unhealthy Phenotype as Indicated by Salivary Biomarkers: Glucose, Insulin, VEGF-A, and IL-12p70 in Obese Kuwaiti Adolescents

**DOI:** 10.1155/2016/6860240

**Published:** 2016-03-16

**Authors:** Mor-Li Hartman, J. Max Goodson, Ping Shi, Jorel Vargas, Tina Yaskell, Danielle Stephens, Maryann Cugini, Hatice Hasturk, Roula Barake, Osama Alsmadi, Sabiha Al-Mutawa, Jitendra Ariga, Pramod Soparkar, Jawad Behbehani, Kazem Behbehani, Francine Welty

**Affiliations:** ^1^Department of Applied Oral Sciences, The Forsyth Institute, Cambridge, MA 02142, USA; ^2^Department of Nutrition, Dasman Diabetes Institute, 15462 Dasman, Kuwait; ^3^Genome Center, The Dasman Diabetes Institute, 15462 Dasman, Kuwait; ^4^Ministry of Health, 13001 Safat, Kuwait; ^5^Faculty of Dentistry, Kuwait University, 13060 Safat, Kuwait; ^6^Dasman Diabetes Institute, 15462 Dasman, Kuwait; ^7^Division of Cardiology, Beth Israel Deaconess Medical Center, Boston, MA 02215, USA

## Abstract

*Objective*. Here, we investigated the relationships between obesity and the salivary concentrations of insulin, glucose, and 20 metabolic biomarkers in Kuwaiti adolescents. Previously, we have shown that certain salivary metabolic markers can act as surrogates for blood concentrations.* Methods*. Salivary samples of whole saliva were collected from 8,317 adolescents. Salivary glucose concentration was measured by a high-sensitivity glucose oxidase method implemented on a robotic chemical analyzer. The concentration of salivary insulin and 20 other metabolic biomarkers was assayed in 744 randomly selected saliva samples by multiplexed bead-based immunoassay.* Results*. Obesity was seen in 26.5% of the adolescents. Salivary insulin predicting hyperinsulinemia occurred in 4.3% of normal-weight adolescents, 8.3% of overweight adolescents, and 25.7% of obese adolescents (*p* < 0.0001). Salivary glucose predicting hyperglycemia was found in only 3% of obese children and was not predictive (*p* = 0.89). Elevated salivary glucose and insulin occurring together was associated with elevated vascular endothelial growth factor and reduced salivary interleukin-12.* Conclusion.* Considering the surrogate nature of salivary insulin and glucose, this study suggests that elevated insulin may be a dominant sign of metabolic disease in adolescent populations. It also appears that a proangiogenic environment may accompany elevated glucose in obese adolescents.

## 1. Introduction

Overweight and obesity among children and adolescents is a global health problem, affecting not only developed nations but also low- to middle-income countries [1]. Childhood obesity is an independent risk factor for adult obesity [2] and has been linked to significant medical, social, academic, and psychological consequences [3]. In all age ranges, obesity is strongly associated with adverse health effects including hypertension, dyslipidemia, insulin resistance, hyperglycemia, and type 2 diabetes [4]. Given these data, it is of critical importance to identify obese children and adolescents with unfavorable metabolic risk factors who are at increased risk of developing comorbidities associated with obesity. Early detection may allow for early intervention, potentially preventing the development of chronic diseases.

Traditionally, studies to identify adverse metabolic risk factors in children have focused on the levels of glucose, insulin, lipids, and/or biomarkers in serum or plasma. However, blood sampling can be particularly difficult for metabolic disease studies involving large cohorts of children, for whom the frequency of adverse reactions related to obtaining blood samples becomes an important limiting factor [5]. In one metabolic study of 12,761 children aged 11 to 13 years, a total of 256 adverse events (2%) that were related to obtaining intravenous blood samples were reported [6]. Most were mild, but 35 instances of loss of consciousness were reported (0.3%). Because of these adverse events, on-site emergency medical personnel were maintained during blood drawing as a precaution. In many types of studies, nonfatal adverse events occurring at a frequency of 2% would be considered acceptable; however, parental consent is a major limiting factor in enrollment for large metabolic studies from which the children derive no direct benefit. Therefore, if blood sampling studies are the only option, many unanswered questions in the field of obesity research will likely remain [5].

The objective of our investigation is to study the development of metabolic diseases in children. To investigate adverse metabolic marker progression in adolescents across body weight categories, we have been conducting studies based on saliva sample analysis rather than on blood sampling. This allows for studies of sufficient size to be performed with a high degree of child acceptance. We have been able to noninvasively collect data from over 8,000 10- to 11-year-old adolescents [7] who live in Kuwait, a country where over half of the adult population is obese [8] and one-quarter of adults have type 2 diabetes [9]. We recently published evidence which confirmed that salivary glucose concentration is significantly correlated with blood glucose concentration in 10-year-old adolescents [10]. The relationship is defined as [plasma concentration = 13.5 × saliva concentration + 84.8], in which 84.8 mg/dL is a salivary glucose threshold concentration. In that analysis, we found that a salivary glucose concentration ≥ 1.13 mg/dL was equivalent to the hyperglycemic blood glucose concentration ≥ 100 mg/dL. In a second study, we analyzed the concentration of several protein biomarkers in saliva samples from a subset of 744 adolescents selected randomly from the larger study population and found that obese adolescents had significantly elevated levels of insulin, C-reactive protein, and leptin, along with significantly reduced levels of adiponectin, when compared to their normal-weight counterparts [7]. In the current report, we have expanded our previous studies by using high-throughput methodology to analyze metabolic markers in 8,245 saliva samples given by the Kuwaiti adolescents across every body weight category. Using this data, we evaluated the relationship between salivary insulin and glucose concentrations and obesity and identified various clinical and biomarker predictors of elevated salivary glucose concentration by body weight category.

## 2. Methods 

### 2.1. Study Population and Design

The Kuwait study population and study design have been previously described [7]. Adolescents enrolled were native Kuwaitis who were in 4th or 5th grade and were approximately equally distributed among each region (governorate) of Kuwait. A total of 8,317 adolescents participated in the study during 182 visits to 138 Kuwaiti schools between October 2, 2011, and May 15, 2012. The study was approved by the Dasman Diabetes Institute Ethical Review Committee in Kuwait. Arabic language informed consent was signed by parents/guardians in advance. Participant assent was obtained from the adolescents on the day of their evaluation.

### 2.2. Salivary Glucose Concentration Analysis

Approximately 3 mL of saliva was collected from each child in a ≥6-hour fasting state, as described previously [7]. The mean salivary flow rate was 26 ± 15.6 mL/hour (0.43 mL/min), such that, on average, a 3 mL sample was collected in 6.9 minutes. Saliva samples were transported on ice from the collection site to the Tissue Bank Core Facility in the Dasman Institute for storage and analysis.

All saliva samples were weighed to determine their volume and centrifuged at 2,800 RPM at 4°C for 20 minutes to remove particulate debris and exfoliated mucosal cells. For salivary glucose determination, 400 *μ*L of the supernatant was transferred to a screw cap 2D barcoded storage tube (Matrix*™* tube, Thermo Fisher Scientific Inc., Waltham, Massachusetts, USA), and the tube was read by a barcode reader (Thermo Scientific VisionMate ST, Thermo Fisher Scientific Inc., Waltham, Massachusetts, USA). During sample preparation, the barcode was captured with subject number to a spreadsheet. The tubes were sealed by a torque-controlled tube capper (Thermo Scientific 8-Channel Screw Cap Tube Capper, Thermo Fisher Scientific Inc., Waltham, Massachusetts, USA) and placed in a 96-vial rack (Thermo Scientific Latch Rack, Thermo Fisher Scientific Inc., Waltham, Massachusetts, USA). Because the glucose assay used a high-sensitivity fluorescent method involving a peroxide intermediate, the capped 96-vial rack was boiled for 1 hour without loss of volume to denature the peroxidase and catalase known to be endogenously present in saliva [11–13]. After boiling, sample racks were maintained frozen at −80°C. Samples were air-transferred from Kuwait under temperature-monitored dry ice (Biocair, Boston, Massachusetts, USA) to the Forsyth Institute and were maintained at −80°C until assay (average time to assay * *= * *0.88 ± 0.06 y).

The measurement of salivary glucose was by fluorescent assay (Glucose Colorimetric/Fluorometric Assay Kit #K606-100, BioVision, Inc., Mountain View, CA, USA) implemented on a Tecan Freedom EVO® 150 robotic processor with an 8-channel liquid handling arm (Tecan Group Ltd., Männedorf, Switzerland). By this assay, an enzyme mixture specifically oxidizes glucose to generate hydrogen peroxide, which then reacts with a dye to generate color (*λ* = 570 nm) and fluorescence (Ex/Em = 535/587 nm). The fluorescence was measured by a spectrophotometer (Infinite® 200 Pro, Tecan Group Ltd., Männedorf, Switzerland) using reverse 96-well plate reading mode. The 3-sigma detection limit of the glucose assay was 0.002 mg/dL. Thirty microliters of saliva supernatant was assayed for each sample. Standards of 0.12, 0.24, 0.48, and 0.96 mg/dL were assayed in triplicate on each run. Upon testing, samples that had been stored at −80°C for varying periods of time up to one year were found to undergo a slow but significant reduction in glucose concentration (Figure S1 in Supplementary Material available online at http://dx.doi.org/10.1155/2016/6860240) at a rate of 0.242 mg/dL/year (*r* ^2^ = 0.55, *p* < 0.0001). Since the assay time and collection time of each of our samples were recorded, we were able to accurately compute the elapsed time and correct for this temporal degradation by adding the computed value to measured values. Corrected values are used in this report.

### 2.3. Analysis of Salivary Biomarkers

The salivary concentration of 20 biomarkers was evaluated using multiplex assays on a random cohort of 744 saliva samples taken from the larger population of 8,245 saliva samples, as described previously [7]. The 20 biomarkers included insulin, interferon-*γ* (IFN-*γ*), interleukin-10 (IL-10), IL-12p70, IL-13, IL-17A, IL-1*β*, IL-4, IL-6, IL-8, monocyte chemotactic protein-1 (MCP-1), tumor necrosis factor-*α* (TNF-*α*), vascular endothelial growth factor-A (VEGF-A), ghrelin, leptin, myeloperoxidase, matrix metalloproteinase-9 (MMP-9), adiponectin (total), C-reactive protein (CRP), and resistin.

### 2.4. Statistical and Analytical

Body weight categories used in the analyses were those defined by the World Health Organization (WHO) using a body mass index (BMI)* Z*-score [14]. By this criterion, “obese” was ≥95th percentile, “overweight” was ≥85th to <95th percentile, “normal weight” was ≥5th to <85th percentile, and “underweight” was <5th percentile. Hypertension was defined as ≥130 mm Hg systolic or ≥85 mm Hg diastolic blood pressure [15]. High salivary insulin was defined as >177 pg/mL (equivalent to approximately 15 *μ*U/mL in blood) [16]. High salivary glucose was defined as ≥1.13 mg/dL (equivalent to approximately ≥100 mg/dL in blood) [10].

To evaluate the relationship between salivary glucose concentration and salivary biomarker concentrations, we used stepwise selection to develop a parsimonious linear model to predict saliva glucose level (SAS 9.3, Proc Glmselect). Because the distribution of glucose was skewed, glucose concentration was log-transformed so that it could be reasonably fit by a linear model, and the values of biomarkers were standardized prior to analysis. During the selection process, all biomarkers and variables of subject characteristics were considered, and Schwartz Bayesian Criterion was evaluated for all models obtained by deleting a variable from the current model or adding a variable to this model. To avoid overfitting, a 10-fold cross validation procedure was adopted to assess the performance, and the best model was determined based on the averaged predictive performance in test sets. For further analysis, this best model was applied to subgroups stratified by WHO body weight categories, with adjustment for additional covariates including age, BMI, and region (SAS 9.3, Proc Glmselect).

## 3. Results

### 3.1. Demographic Data

We enrolled 8,317 adolescents with a mean age of 10 ± 0.7 y in this study. The majority of adolescents were female (61.2%). The average waist circumference was 68.7 ± 19.4 cm, and the average BMI of the population was 20.9 ± 5.3 kg/m^2^. The clinical characteristics based on body weight categories (underweight, normal weight, overweight, and obese) are shown in Table 1. About one-quarter (26.5%) of the adolescents were obese.

### 3.2. Salivary Insulin Concentration by Body Weight Category

Given that insulin resistance and hyperinsulinemia are well-known features of the progression to a metabolically unhealthy obese phenotype in adolescents [17], we evaluated the salivary insulin concentrations from a cohort of 744 adolescents that were randomly selected from the larger study population across all body weight categories. It has been suggested that fasting blood insulin levels greater than 15 mU/L may be useful for evaluating insulin resistance [16]. Using the relationship we have previously established between blood and salivary levels of insulin [7], a correspondingly high insulin level in saliva would be ≥177 pg/mL. With this value, we found that the prevalence of high salivary insulin concentration, indicating a state of insulin resistance and hyperinsulinemia, was increased in obese adolescents. None of the underweight adolescents, 4.3% of the normal-weight adolescents, 8.3% of the overweight adolescents, and 25.7% of the obese adolescents had elevated salivary insulin concentrations (*p* < 0.0001).

### 3.3. Salivary Glucose Concentration by Body Weight Category

Continuing insulin resistance and hyperinsulinemia can lead to hyperglycemia [18], which is a hallmark of type 2 diabetes and the metabolic syndrome. Therefore, we analyzed glucose concentration in the saliva samples from the larger study population. As we have previously demonstrated, blood hyperglycemia (≥100 mg/dL) is approximated by the salivary glucose level of ≥1.13 mg/dL [10]. We were able to measure salivary glucose concentrations in 8,245 (99.1%) of the saliva samples; 72 samples (0.9%) were missing. The loss of samples was due to mechanical malfunction. The average salivary glucose concentration was 0.19 ± 0.24 mg/dL (range: 0–3.5 mg/dL). Interestingly, there were no significant differences in the average salivary glucose concentration between body weight categories.

**Figure 1:**
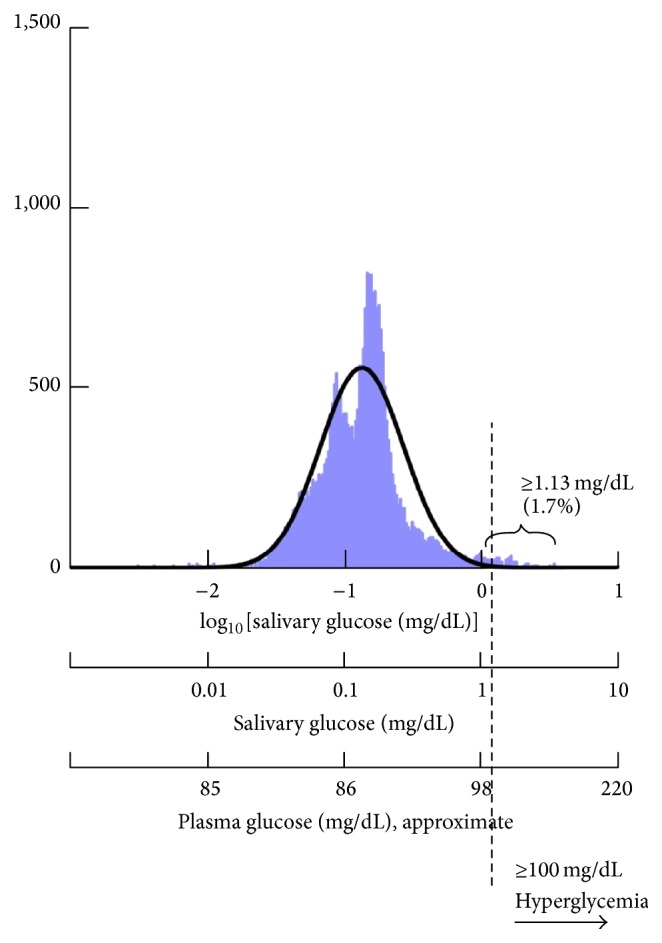
Semilogarithmic distribution of salivary glucose concentration values. Approximate values of the concentration of plasma glucose were computed from the equation [plasma = 13.5 × saliva + 84.8] [10]. The salivary glucose concentration of 1.13 mg/dL was computed from the same equation and is approximately equivalent to a hyperglycemic plasma value of 100 mg/dL.

Among the subset of 744 adolescents randomly selected across all weight categories, overall 1.7% had salivary glucose levels suggestive of hyperglycemia (≥1.13 mg/dL) (Figure 1). By weight category, salivary glucose levels were suggestive of hyperglycemia seen in none of the underweight adolescents, 1.1% of the normal-weight adolescents, 0.8% of the overweight adolescents, and 3.0% of the obese adolescents (*p* = 0.89).

### 3.4. Salivary Glucose in Type 1 Diabetic Children

The validity of salivary diagnosis was suggested by a response to the question, “Do you have diabetes?” Thirty answered “yes” and also indicated that they were taking insulin. These children (presumably type 1 diabetics) had average salivary glucose of 0.27 ± 0.39. The other 8,138 children answered “no” with an average salivary glucose level of 0.19 ± 0.24. This difference was significant (*p* = 0.013) suggesting that salivary glucose was higher in children diagnosed with type 1 diabetes.

### 3.5. Relationship between Salivary Insulin and Glucose Concentrations and Obesity

**Figure 2:**
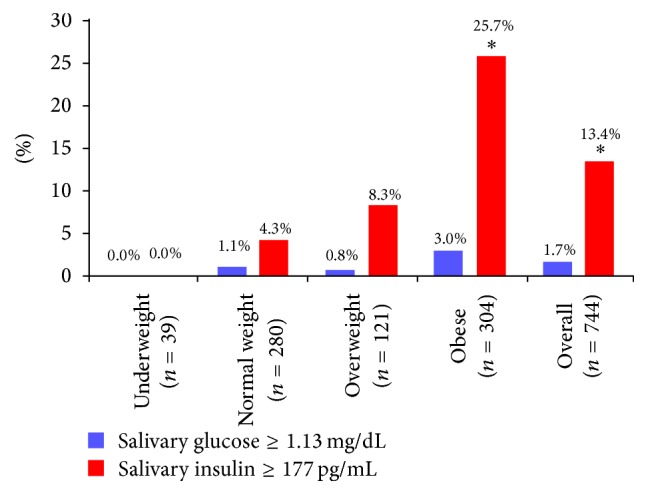
Percentage of the subset of 744 adolescents in each body weight category who had high salivary insulin and glucose concentrations. Only the values marked with an asterisk differed between high glucose and high insulin for each body weight category.

We next compared the proportion of adolescents who had high salivary insulin concentration with the proportion of adolescents with high salivary glucose concentration among the 744 adolescents in the randomly selected cohort by body weight category (Figure 2). We found that while the prevalence of high salivary glucose and insulin concentrations both increased with obesity, they did not increase proportionally. About 25% of the obese adolescents were hyperinsulinemic but only 3.0% were hyperglycemic by our salivary analysis. In other words, in obese adolescents, the prevalence of high salivary glucose concentration increased only 3-fold, while the prevalence of high salivary insulin concentration increased 6-fold, as compared to normal-weight adolescents. A logistic binary regression analysis indicated that the odds ratio for elevated salivary glucose concentration in obese adolescents was 2.44 (95% CI: 0.69–8.67) and for elevated salivary insulin concentration was 6.41 (95% CI: 3.88–10.58). Therefore, in our cohort, obesity was significantly related to elevated salivary insulin concentration but not to elevated salivary glucose concentration.

### 3.6. Predictors of Elevated Salivary Glucose Concentration by Body Weight Category

Hyperglycemia is among the key factors indicating progression to a metabolically unhealthy obese phenotype in adolescents [19]. A simple analysis of correlation between salivary glucose and salivary biomarkers reveals that, of the 20 biomarkers measured, only insulin, VEGF, and IL-12p70 were significantly associated with each other (Table 2). To better understand the risk factors involved in the development of adolescent hyperglycemia, we built a predictive model for log-transformed salivary glucose levels that considered both clinical variables and salivary biomarker concentrations. The concentrations of 20 salivary biomarkers, including inflammatory markers and adipocytokines, were assayed in saliva samples from the cohort of 744 adolescents that were randomly selected from the larger study population across all body weight categories. Clinical variables from the larger study population were used for the benefit of a large sample size (*n* = 8,245). A data-driven stepwise selection procedure with 10-fold cross validation yielded a parsimonious model containing sex, insulin, VEGF-A, and IL-12p70 as significant predictors of salivary glucose levels (Table 3). None of the other clinical variables nor any of the other 17 biomarkers in saliva that were tested qualified for this analysis.

Further analysis was conducted in subgroups by body weight category. In each group, a predictive model for salivary glucose concentration was built including the above selected predictors, as adjusted for age, BMI, and region. Among the clinical variables, the geographic region of Kuwait (governorate) was a significant factor in salivary glucose for all body weight classes except underweight. Participant sex was a significant predictor for salivary glucose concentration in the normal body weight group (*p* = 0.006) and the overweight group (*p* = 0.004).

Among the salivary biomarkers, none was a significant predictor for salivary glucose concentration in the underweight group. Salivary insulin concentration was a significant predictor for salivary glucose concentration in the overweight (*p* = 0.02) group. In the obese group, the salivary concentrations of insulin, VEGF-A, and IL-12p70 were significant predictors for salivary glucose concentration, with insulin being the most robust (*p* < 0.0001), followed by VEGF-A (*p* = 0.01) and IL-12p70 (*p* = 0.01). Both insulin and VEGF-A were positively associated with salivary glucose concentration, while IL-12p70 showed an inverse association.

## 4. Discussion

Insulin resistance is a major factor in obesity-related, deleterious metabolic changes. Attenuated actions of insulin result in hyperglycemia, decreased protein synthesis, increased protein degradation, and increased susceptibility to infection [18]. Our current analysis of saliva samples from 8,245 Kuwaiti 10-year-old adolescents suggests that obesity in this population is significantly associated with high salivary insulin concentration but not with high salivary glucose concentration. Others have observed that hyperinsulinemia though often seen in children during puberty is not generally included as a risk factor for metabolic disease [20]. It has been suggested that fasting plasma insulin levels >15 mU/L could be a reasonable clinical alternative for evaluating insulin resistance [16]. A very small percentage of adolescents in this study (1.7%) had fasting salivary glucose levels that would be considered hyperglycemic (≥1.13 mg/dL in saliva, equivalent to ≥100 mg/dL in plasma), and only 26.9% of those adolescents were obese. In contrast, a much larger percentage of the adolescents (13.4%) had fasting hyperinsulinemia (≥177 pg/mL in saliva, equivalent to ≥15 mU/L in plasma), and 78% of those adolescents were obese. This observation supports the idea that a state of developing insulin resistance is present in these adolescents. The extraordinary differences in the fold increase of fasting salivary insulin concentration compared with fasting salivary glucose concentration in obese and overweight adolescents, however, suggest there may be factors outside the insulin-glucose control loop [21, 22] that accentuate insulin release. Exogenous stimulation of insulin release and resultant hypoglycemic episodes could be a reasonable mechanism by which obesity occurs in these adolescents.

In considering the progression from hyperinsulinemia to hyperglycemia, we conducted a salivary concentration analysis for 20 different biomarkers to determine their correlation with elevated salivary glucose concentration. VEGF-A was one biomarker found to be positively correlated with salivary glucose concentration. VEGF-A is widely recognized as a signal protein that stimulates angiogenesis. Infusion of glucose alone has been shown to increase plasma VEGF levels [23], and elevated blood glucose in obese children has been associated with increased blood levels of VEGF-A [24]. Therefore, it is reasonable to assume that the process of developing increased body mass would be associated with an increased need for vascularization and, hence, increased levels of VEGF-A. It is also possible that the early association of increased VEGF-A levels with obesity and hyperglycemia could be related to the development of diabetic renal disease and/or retinopathy [25].

Interestingly, IL-12p70 levels in saliva were inversely correlated to salivary glucose levels. In addition to its ability to stimulate differentiation of native T cells into Th1 cells, IL-12p70 is also antiangiogenic [26]. Since the obese environment has both elevated levels of glucose and VEGF-A, supporting angiogenesis in a growing body mass condition, inhibition of IL-12p70 could be part of this milieu. Indeed, it has been suggested that balance between pro- and antiangiogenic cytokines may be one of the factors that prevents diabetic kidney and retinal disease [27]. Further studies will be needed to elucidate the role of IL-12p70 in the development of hyperglycemia, insulin resistance, and obesity.

In summary, we observed that obesity in Kuwaiti adolescents was significantly related to high salivary insulin concentration but not to high salivary glucose concentration. For those adolescents who did have salivary glucose concentrations suggesting hyperglycemia, high salivary glucose concentrations were associated with increased levels of salivary insulin and VEGF-A and reduced salivary levels of IL-12p70. The current study expands our previous validation studies for glucose [10] and insulin [7] that indicated a significant correlation between plasma or serum concentrations and salivary concentrations of these molecules. Other investigators have presented data that indicate that salivary concentrations of VEGF-A [28] and IL-12p70 [29] are also significantly correlated (*r* = 0.65, 0.34, resp.) with blood levels. Although we can only speculate at this stage, it seems that we are able to detect early changes in salivary biomarkers in 10-year-old adolescents that could suggest the initiation of deleterious metabolic changes associated with obesity. Follow-up studies will focus on a wider selection of biomarkers to study in saliva to more precisely characterize these early changes. We will also expand our current observations to different populations of adolescents in order to increase clinical relevance.

Salivary concentrations of insulin and glucose can act as surrogates for their serum or plasma concentrations. We found that hyperinsulinemia may be more prominent than hyperglycemia in the early stages of obesity-related metabolic disease, and elevated insulin may be a dominant sign of metabolic disease. When hyperglycemia occurs in adolescents, it may be accompanied by a proangiogenic environment.

## Supplementary Material

Supplementary Figure 1. When saliva samples were stored under refrigeration (-80°C), a reduction in measured glucose concentration was observed. The following figure illustrates the change in salivary glucose concentration observed over a 1 year period. The associated function ?G=0.242 x t(years) was used to correct all measured glucose values.

## Figures and Tables

**Table 1:** Demographic data and clinical characteristics of 8,317 enrolled children overall and by weight category.

	Body weight category				Overall
	Underweight	Normal weight	Overweight	Obese	
*N* (%)	193 (2.3)	4,129 (49.6)	1,791 (21.5)	2,204 (26.5)	8,317 (100)
Age (y)^a^	9.8 ± 0.6	9.9 ± 0.7	10.0 ± 0.7	10.1 ± 0.7	10.0 ± 0.7
Waist circumference (cm)^b^	54.3 ± 5.1	60.4 ± 10.1	70.2 ± 7.1	84.1 ± 28.3	68.7 ± 19.4
BMI (kg/m^2^)^b^	13.5 ± 0.8	17.1 ± 1.6	21.7 ± 1.3	27.9 ± 4.3	20.9 ± 5.3
Salivary glucose concentration (mg/dL)	0.20 ± 0.25	0.20 ± 0.25	0.18 ± 0.22	0.20 ± 0.26	0.19 ± 0.24
Salivary glucose ≥ 1.13 mg/dL, *n* (%)	4 (2.1)	72 (1.7)	27 (1.5)	38 (1.7)	141 (1.7)
Saliva flow rate (mL/h)^c^	22.8 ± 12.4	24.9 ± 14.5	26.8 ± 15.7	27.8 ± 17.2	26.0 ± 15.6
Diastolic blood pressure (mm Hg)^a^	69.2 ± 12.4	70.2 ± 16.2	75.5 ± 19.3	81.3 ±27.0	74.3 ± 20.7
Systolic blood pressure (mm Hg)^a^	101.1 ± 13.9	103.3 ± 14.8	112.3 ± 14.4	119.4 ± 14.8	109.4 ± 16.3
Hypertension, *N* (%)^a^	26 (13.5)	574 (13.9)	426 (23.8)	964 (43.8)	1990 (23.9)

^a^All significantly different except for “underweight” and “normal weight” (*p* < 0.001).

^b^All categories significantly different (*p* < 0.001).

^c^All categories significantly different except for “overweight” compared to “obese” (*p* < 0.001).

**Table 2:** Correlation coefficients (*r*) with *p* values between salivary glucose and salivary biomarkers sorted by ascending order of *p*.

Biomarker	Correlation coefficient		Biomarker	Correlation coefficient	
	*r*	*p*		*r*	*p*
Insulin	0.254	<0.0001	IL-10	−0.066	0.255
VEGF	0.162	0.005	IL-8	0.063	0.275
IL-12p70	−0.121	0.035	MCP-1	0.058	0.312
MPO	−0.109	0.059	IL-4	−0.053	0.354
IL-13	−0.092	0.108	Leptin	0.051	0.377
IL-17A	−0.089	0.121	IL-1*β*	−0.043	0.452
Ghrelin	−0.087	0.132	Adiponectin	−0.037	0.517
TNF-*α*	−0.084	0.145	IL-6	−0.035	0.548
Resistin	−0.078	0.176	CRP	−0.033	0.561
IFN-*γ*	−0.071	0.215	MMP-9	0.011	0.845

**Table 3:** Analysis of the association between salivary glucose, demographic factors, and biomarkers in a subset of 744 children stratified by body weight category. Values marked with an asterisk are statistically significant (*p* < 0.05).

	Underweight (*n* = 39)		Normal (*n* = 280)		Overweight (*n* = 121)		Obese (*n* = 304)	
	Estimates (SE)	*p* value	Estimates (SE)	*p* value	Estimates (SE)	*p* value	Estimates (SE)	*p* value
Age (per year)	−0.26 (0.18)	0.16	−0.07 (0.08)	0.37	−0.07 (0.13)	0.52	0.02 (0.07)	0.84
Sex (boys versus girls)	0.23 (0.29)	0.43	−0.26 (0.09)	0.006^*∗*^	−0.49 (0.14)	0.004^*∗*^	−0.09 (0.10)	0.34
BMI (per unit)	0.16 (0.17)	0.35	−0.02 (0.03)	0.42	0.06 (0.07)	0.4	−0.01 (0.01)	0.54
Insulin (per SD)	0.52 (0.73)	0.48	0.16 (0.08)	0.06	0.25 (0.11)	0.02^*∗*^	0.15 (0.04)	<0.0001^*∗*^
VEGFA (per SD)	0.16 (0.18)	0.38	0.07 (0.06)	0.26	−0.08 (0.12)	0.53	0.10 (0.04)	0.01^*∗*^
IL-12p70 (per SD)	−0.06 (0.13)	0.66	−0.07 (0.05)	0.13	−0.005 (0.06)	0.93	−0.14 (0.05)	0.01^*∗*^
Governorate		0.12^a^		<0.0001^a,*∗*^		<0.0001^a,*∗*^		<0.0001^a,*∗*^

^a^ *p* value was for all governorates in a type 3 analysis.

## Abbreviations

BMI: Body mass index
IFN: Interferon
IL: Interleukin
MMP-9: Matrix metalloproteinase-9
RPM: Revolutions per minute
TNF-*α*: Tumor necrosis factor-*α*
WHO: World Health Organization
VEGF-A: Vascular endothelial growth factor-A.
