# Supplementary material for: Unhealthy Phenotype as Indicated by Salivary Biomarkers: Glucose, Insulin, VEGF-A, and IL-12p70 in Obese Kuwaiti Adolescents
Source: J Obes. 2016 Mar 16;2016:6860240. doi: 10.1155/2016/6860240 (PMC4812454; doi:10.1155/2016/6860240)
Supplement: Supplementary file 1 — Supplementary Figure 1. When saliva samples were stored under refrigeration (-80°C), a reduction in measured glucose concentration was observed. The following figure illustrates the change in salivary glucose concentration observed over a 1 year period. The associated function ?G=0.242 x t(years) was used to correct all measured glucose values. [file 6860240.f1.docx]

**
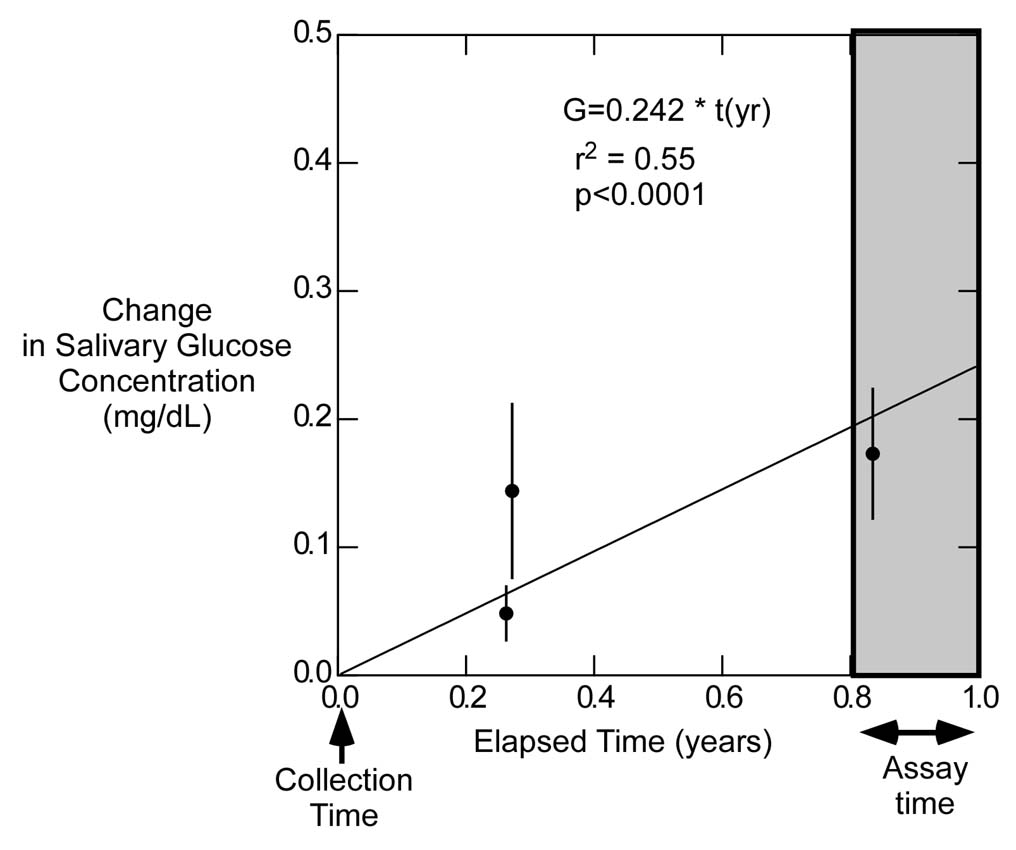
**

**Supplementary Figure 1**.

When saliva samples were stored under refrigeration (-80°C), a reduction in measured glucose concentration was observed. The following figure illustrates the change in salivary glucose concentration observed over a 1 year period. The associated function ?G=0.242 x t(years) was used to correct all measured glucose values.
